# Machine learning with the TCGA-HNSC dataset: improving usability by addressing inconsistency, sparsity, and high-dimensionality

**DOI:** 10.1186/s12859-019-2929-8

**Published:** 2019-06-17

**Authors:** Michael C. Rendleman, John M. Buatti, Terry A. Braun, Brian J. Smith, Chibuzo Nwakama, Reinhard R. Beichel, Bart Brown, Thomas L. Casavant

**Affiliations:** 1 Department of Electrical and Computer Engineering, Center for Bioinformatics and Computational Biology, University of Iowa, 5017 Seamans Center, Iowa City, IA 52242 USA; 2 Department of Radiation Oncology, Carver College of Medicine, University of Iowa Carver College of Medicine, LL-W Pomerantz Family Pavilion, 200 Hawkins Drive, Iowa City, IA 52242-1089 USA; 3 Department of Biomedical Engineering, Center for Bioinformatics and Computational Biology, University of Iowa, 5017 Seamans Center, Iowa City, IA 52242 USA; 4 Department of Biostatistics, University of Iowa, 145 N. Riverside Drive, 100 CPHB, Iowa City, IA 52242 USA; 5 Center for Bioinformatics and Computational Biology, University of Iowa, 5017 Seamans Center, Iowa City, IA 52242 USA; 6 Iowa Institute for Biomedical Imaging, Department of Electrical and Computer Engineering, Department of Internal Medicine, The University of Iowa, Iowa City, 52242 IA USA; 7 3312 Seamans Center for the Engineering Arts and Sciences, Iowa City, IA 52242-1527 USA

**Keywords:** Machine learning, hnscc, tcga, Dimensionality reduction, Gene ontology enrichment analysis, Decision support, Unsupervised transformation

## Abstract

**Background:**

In the era of precision oncology and publicly available datasets, the amount of information available for each patient case has dramatically increased. From clinical variables and PET-CT radiomics measures to DNA-variant and RNA expression profiles, such a wide variety of data presents a multitude of challenges. Large clinical datasets are subject to sparsely and/or inconsistently populated fields. Corresponding sequencing profiles can suffer from the problem of high-dimensionality, where making useful inferences can be difficult without correspondingly large numbers of instances. In this paper we report a novel deployment of machine learning techniques to handle data sparsity and high dimensionality, while evaluating potential biomarkers in the form of unsupervised transformations of RNA data. We apply preprocessing, MICE imputation, and sparse principal component analysis (SPCA) to improve the usability of more than 500 patient cases from the TCGA-HNSC dataset for enhancing future oncological decision support for Head and Neck Squamous Cell Carcinoma (HNSCC).

**Results:**

Imputation was shown to improve prognostic ability of sparse clinical treatment variables. SPCA transformation of RNA expression variables reduced runtime for RNA-based models, though changes to classifier performance were not significant. Gene ontology enrichment analysis of gene sets associated with individual sparse principal components (SPCs) are also reported, showing that both high- and low-importance SPCs were associated with cell death pathways, though the high-importance gene sets were found to be associated with a wider variety of cancer-related biological processes.

**Conclusions:**

MICE imputation allowed us to impute missing values for clinically informative features, improving their overall importance for predicting two-year recurrence-free survival by incorporating variance from other clinical variables. Dimensionality reduction of RNA expression profiles via SPCA reduced both computation cost and model training/evaluation time without affecting classifier performance, allowing researchers to obtain experimental results much more quickly. SPCA simultaneously provided a convenient avenue for consideration of biological context via gene ontology enrichment analysis.

## Background

Data generated for standard clinical oncology care has expanded exponentially. In addition to well-known clinical variables like symptoms, stage and histology, tumor specimens are now routinely sequenced for a range of mutations that may be more or less well characterized. These molecular profiles may suggest sensitivity to a range of molecularly targeted agents. Furthermore, high resolution, functional and molecular imaging methods (like CT-PET and MR) create quantitative metrics described through radiomics features. These also suggest profiles that can guide intervention and response. To facilitate the development of novel clinical decision support tools for oncologists, we have used the publicly available data characterizing head and neck squamous cell carcinoma (HNSCC). These profiles present a large data analysis problem necessitating the use of machine learning, dimensionality reduction, and biological pathway analysis techniques. We utilize machine learning classifiers to predict patient two-year recurrence-free survival and evaluate a variety of feature sets to discover potential useful clinical biomarkers. Feature sets include combinations of patient clinical and molecular data. To improve utility of this dataset for oncological decision support, imputation and dimensionality reduction methods are used to transform feature sets to more usable, interpretable forms.

### Existing literature

Current HNSCC literature often focuses on association of regulation of specific genes with prognosis [1, 2]. Other groups, however, acknowledge the need for large-scale integrative analysis to capture potential novel biomarkers [3–5]. In other cancers, unsupervised transformations of molecular data (e.g. RNA sequencing, DNA methylation, miRNA sequencing) are known to be useful in machine learning-based survival prediction [6, 7]. As of this writing, little work has been done with HNSCC in this manner. Literature on machine learning imputation of sparse clinical data is similarly unavailable.

### Dataset

The Cancer Genome Atlas (TCGA) Research Network [8] is a coordinated effort to gather, share, and analyze next generation molecular sequencing data to improve our understanding of cancer mechanisms on a molecular level [9]. Data utilized in our analysis were obtained from the National Cancer Institute Genomic Data Commons Data Portal [10] and contained 528 TCGA-HNSC cases, including genotyping, solid-tumor RNA expression, whole exome sequencing, methylation data, and clinical information. In this work, only RNA expression variables and clinical information are considered. Clinical data includes tumor grading information, patient demographic data, smoking/alcohol histories, and several features related to disease progression such as lymphovascular invasion and margin status. Human papillomavirus (HPV) status (based on ISH and P16 testing) was also included, as HPV status has strong implications for prognosis and tumor development [11, 12]. These data have been contributed from a number of studies from varying institutions, utilizing multiple platforms and assays that span significant time intervals. The work presented here addresses the challenges presented by this common form of dataset in oncological research.

Large, multi-institutional datasets present a variety of challenges to the development of methods and tools for clinical decision support. Namely, several clinical data fields in TCGA-HNSC offered issues of sparsity and inconsistency. Out of 15 identified clinical characteristics relevant to treatment regimen, none were populated for every patient. More specifically, the number of cases (from a total possible 528 cases in TCGA) with missing or unavailable data for these fields ranged from 88 to 504, with a mean of 349.5 and median of 342 cases lacking data for each field. In addition to the problem of missing data, several fields were populated inconsistently, with responses varying both due to human error (e.g. leading zeros in numeric fields) and varying convention (e.g. “External” vs. “EXTERNAL BEAM”). Such complications required extensive preprocessing and an expert system built using domain-specific knowledge to determine whether each patient had received a specific type of therapy. Even after this preprocessing and condensing of treatment fields, issues of missing data persisted. Whether a patient had received radiotherapy and/or chemotherapy was unclear for 47 and 27% of cases, respectively. One possible technique for handling such problems is to exclude cases or variables with missing data, as was done previously with this dataset [13]. Due to the relevance of these features to our decision support goals, as well as the limited number of cases from which to draw, we attempt to maximize utilization of the available data by imputing missing values.

Molecular datatypes are often extremely high-dimensional. Feature selection and dimensionality reduction techniques are necessary steps when utilizing such data to best employ available computational resources. There are several strategies for selection and dimensionality reduction, including feature filtering, feature transformations, and wrapper methods such as sequential selection [14]. In this work, feature filtering and an unsupervised sparse PCA feature space transformation of 20,531 solid-tumor RNA expression variables were employed and evaluated in the context of TCGA-HNSC.

## Methods

### Preprocessing, condensing, and missing data imputation (Fig. 1a)

Performing imputation on large datasets requires development of an expert model. Careful examination and correction of inconsistent and missing values was performed in collaboration with expert oncologist Dr. John Buatti (Chair of Radiation Oncology, University of Iowa). A rubric for consistent preprocessing and condensing of the 15 relevant treatment fields resulted in a much more concise and usable dataset. However, a significant fraction of the TCGA-HNSC patients still had uncertain status in their treatment regimens. To address this, Multivariate Imputation by Chained Equations (MICE) [15] was utilized, and the resulting changes to classifier performance were measured.

**Fig. 1:**
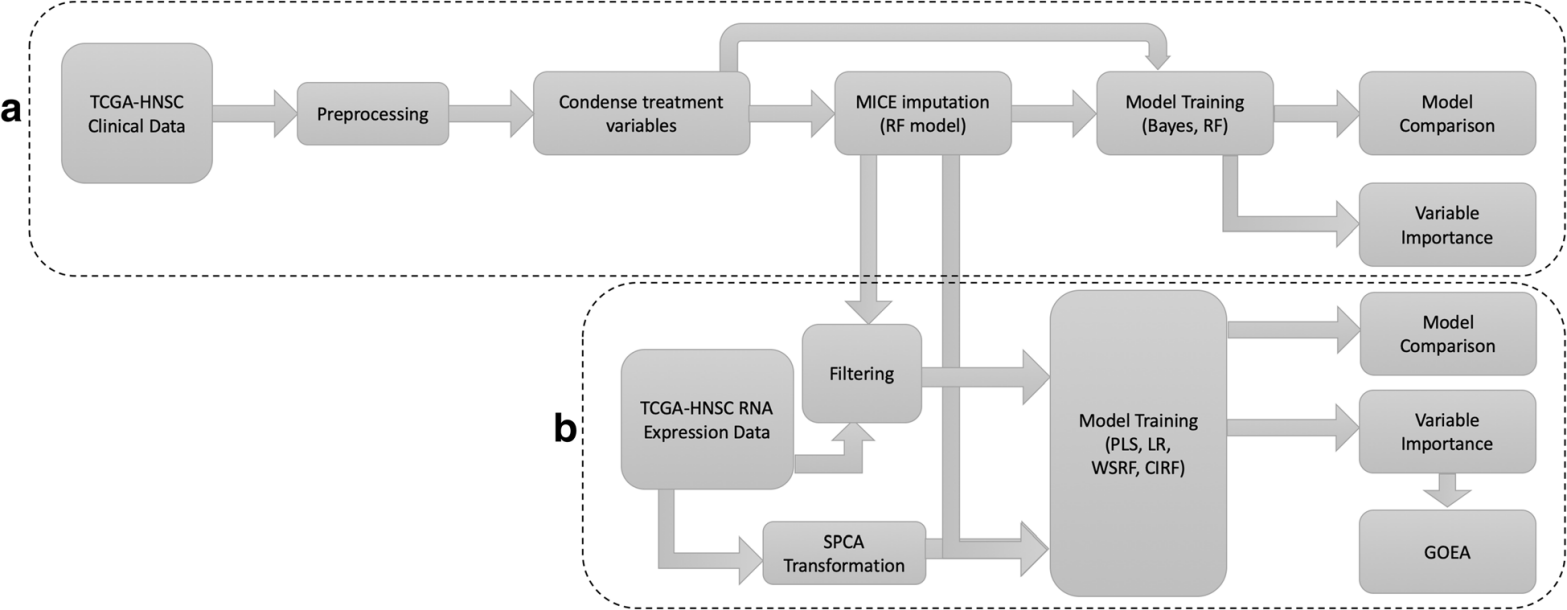
Methods Flowchart. Flow diagram outlining the methods of this work. **a** Clinical data preprocessing and imputation. **b** RNA expression experiments and analysis

MICE builds predictive models for each missing variable to realistically impute entries based on the remaining predictors. It takes into account uncertainty during imputation, allowing it to outperform single imputation methods [16, 17]. For each missing entry, five intermediate imputations were performed using random forest models. Models were trained using 21 clinical characteristics, including grading, metastasis, tobacco usage, HPV status, and other demographic data. Patient outcomes were excluded from the imputation to prevent information leakage or overfitting. As the imputed treatment variables are binary, a majority vote was conducted of the five imputations to yield a final imputation. Using the imputed variables, the clinical characteristics utilized in imputation, and the outcome of two-year recurrence-free survival, two types of model were trained on the pre-imputation and post-imputation datasets. Missing values in the pre-imputation set were given a third category, “Unavailable”.

Naïve Bayes and Random Forest (RF) classifiers were selected for this evaluation. The Bayesian model provides a pure conditional-statistical effort to predict survival, though it does not consider interaction effects. The Random Forest model, being a set of recursive partitionings, extensively leverages interaction effects. Earlier work suggests RF models are effective for this classification problem [18]. The pre- and post-imputation models were compared with respect to both predictive performance and variable importance.

### RNA expression experiments (Fig. 1b)

In the TCGA-HNSC dataset, solid-tumor expression was available for 520 of the 528 patients. With a feature set of 20,531 solid-tumor RNA expression variables, seven tumor grading variables, and the random forest-imputed treatment variables, several RF classifiers were trained to predict two-year recurrence-free survival. The classifiers varied in feature sampling and tree construction procedures: a standard RF, a weighted subspace RF (WSRF) [19], and a conditional inference random forest (CIRF) [20]. The WSRF weights randomly sampled variables based on their correlation with the output procedure, increasing the probability that a given tree will sample variables with high univariate correlation to patient survival. The CIRF utilizes a conditional inference procedure for tree construction that aims to eliminate bias in recursive partitioning and reduce computation time with stopping criteria.

With the full set of RNA expression data, the feature set was first refined through two filters: a univariate, near-zero variance filter to remove uninformative features and a multivariate correlation filter to remove features with correlation greater than 0.9. These filters removed several thousand expression variables from the feature set.

**Fig. 2:**
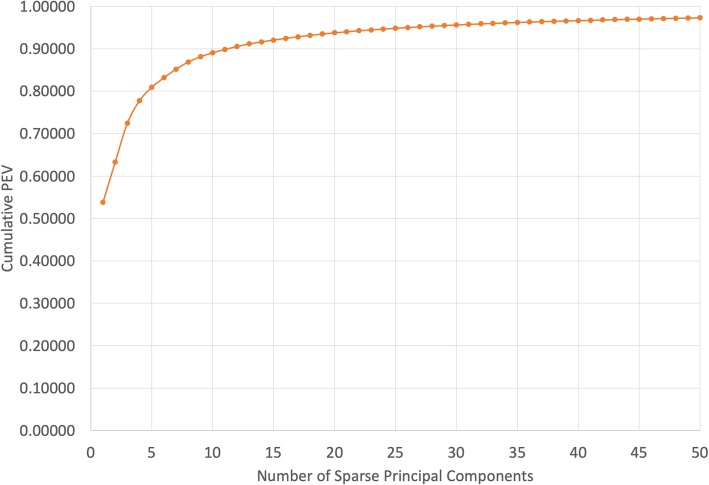
Cumulative Percent Explained Variance. Percent explained variance from SPCA as it relates to the number of components retained. The dark vertical line indicates the value used for transforming RNA expression into the SPCA feature set for these experiments

In addition, a dimensionality reduction was performed via Sparse Principal Component Analysis (SPCA) [21] which has the potential to improve interpretability of the model and reduce training time. Interpretability is improved because each sparse principal component (SPC) has only a handful of genes that contribute to it, allowing connections to be drawn between individual SPCs and the biological processes related to their constituent genes. One significant problem of PCA-based data reduction is choosing the number of components. If too many components are retained, this transformation may be amplifying noise. If too few are included, valuable predictive information may be excluded. To estimate information inclusion, percent explained variance is examined in Fig. 2. Here, we chose the number of principal components to be ten, as this number of components yielded the best classifier performance over the three RF classifiers while explaining approximately 90% of the variance. The resulting ten SPCs (below labeled X1-X10) were constructed and the feature set supplemented with the same grading and treatment features as used with the full set of RNA variables. The same set of RF classifiers was trained on this data to predict two-year recurrence-free survival.

After training, variable importance for the 10-component SPCA feature set was evaluated for each of the classifiers. The genes that comprise the most and least important variables were examined with a gene ontology enrichment analysis (GOEA) [22, 23]. Analysis was conducted with the biological process annotation dataset from the PANTHER Classification System, using their enrichment analysis tool [24]. Gene ontology (GO) terms enriched for high-importance SPC gene sets but not low-importance SPC gene sets are described.

### Classifier training and hyperparameter tuning

A nested cross validation (CV) is used to tune hyperparameters and estimate out-of-sample performance, a measurement of how well a classifier would generalize if it were to be trained on the entire dataset. Data is split into ten folds (or partitions) for the outer CV. In each iteration, one fold acts as the testing data and the remaining nine folds act as the training data. Within each fold of the outer CV, a repeated grid search CV procedure [25] is carried out on the training data to estimate the best hyperparameter(s). Then, a model is trained on all of the training data with the best hyperparameter set, and its generalization performance is estimated using the testing data for that fold. The classifier’s ability to accurately predict the class labels of test data points is then estimated from performance within the ten folds, using a classifier performance metric.

For the missing data imputation, models were trained and tested in Weka 3.9.1 [26] with 10-fold cross validation as the internal cross validation procedure using the “CVParameterSelection” wrapper method. One hyperparameter, the number of randomly chosen predictors to be considered for each split, was tuned for the Random Forest.

In the RNA expression experiments, three different RF classification procedures are considered. Classifier training and evaluation was handled using the R package caret [27]. Classifiers trained on the full-RNA data were evaluated with the internal cross validation procedure as ten-fold cross validation, and those trained with the dimensionality-reduced data were evaluated (within each fold) using five-times repeated ten-fold cross validation. Repeated cross validation reduces bias due to random partitioning [28]. Preprocessing was handled within each CV iteration with the R recipes package [29]. For these RF models, the number of randomly-sampled predictors for each tree was varied over a span of values appropriate for each feature set.

### Classifier performance metric

To evaluate classifier out-of-sample performance, the area under the receiver operating characteristic curve (commonly denoted AUC) is employed. AUC is a very popular and commonly used classifier metric in the literature with an intuitive probabilistic interpretation: AUC is the probability that the classifier will score positive observations higher than negative observations. Mathematically, an AUC of 0.5 is equivalent to random guesses and is the standard baseline for this metric.

### Variable/feature importance

Feature importance is a measurement of how perturbations to variables affect classifier performance. A conditional variable importance procedure has been applied in this work. Conditional importance involves not only univariate perturbations, but conditional perturbation of variables and the variables with which they correlate [30]. For the imputation experiments, the correlation threshold was set at 0.2 for computational viability. In analysis of SPCA variables, this threshold was set to 0.05 as the feature space is smaller. Importance of categorical variables can also be biased in this scenario (depending on the number of categories), so a conditional inference random forest model is used to reduce this bias [20]. Reported importance values are relative to the most important variable in each case and were averaged over 50 runs to ensure stability.

## Results

### Imputation evaluation

As shown in Table 1, imputation of treatment fields using MICE yielded no significant change in AUC. Changes in relative importance values can be seen in Table 2.

**Table 1:** Effect of Imputation on Classifier Performance

Classifier	Dataset	AUC
Naïve Bayes	Pre-imputation	0.633 ± 0.077
	Post-imputation	0.675 ± 0.063
Random Forest	Pre-imputation	0.668 ± 0.062
	Post-imputation	0.675 ± 0.063

Classifier performance on the imputed and non-imputed datasets. Baseline AUC is 0.500

**Table 2:** RNA Expression Classifier Performance

Datasets	Classifiers:	RF	WSRF	CIRF
AUCs				
Full RNA		**0.632 ± 0.106**	0.596 ± 0.038	0.629 ± 0.105
SPCA		0.640 ± 0.128	0.626 ± 0.114	**0.658 ± 0.044**
Nested CV Runtimes		–	–	–
Full RNA		**52 h**	185 h	85 h
SPCA		**12 min**	1.9 h	30 min

AUC and approximate runtime values for the RNA expression feature sets. The best value in each row is bolded. Here, runtimes are evaluation times for a given classifier on a given feature set via 10-fold nested cross validation with the internal cross validation procedures as described in Methods. Computations performed on the University of Iowa’s Argon High-Performance Computing cluster

**Fig. 3:**
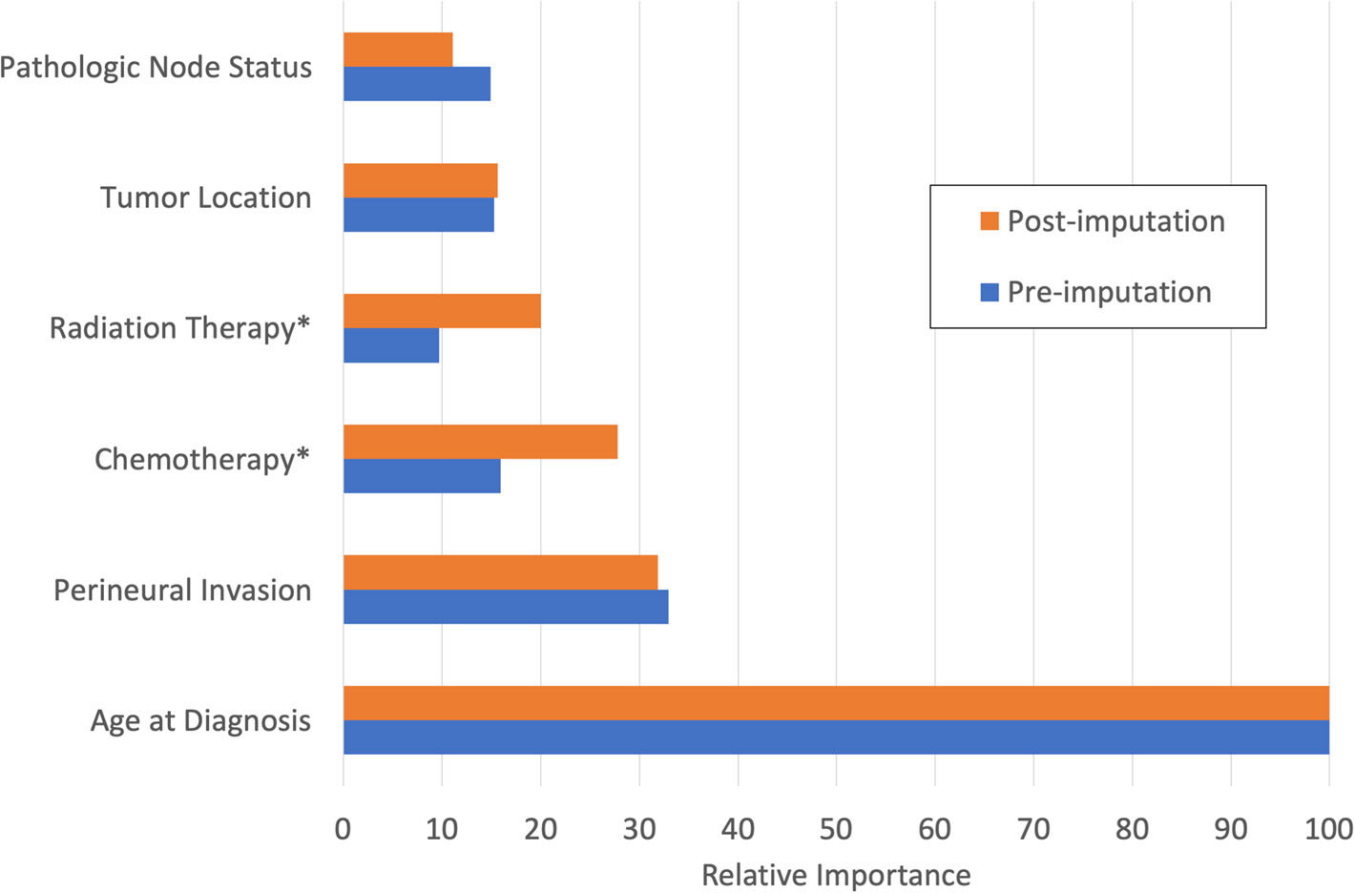
Importance Change with Imputation. Pre-imputation and post-imputation CIRF conditional variable importance for predicting two-year recurrence-free survival. Importance values are relative to the most important variable. Imputed treatment features are denoted with *, and several clinical variables are shown for comparison

The relative importance of treatment features doubled as a result of imputation (see Figure 3). Interestingly, changes were observed in non-imputed features as well, with some features (HPV status, margin status) becoming more important and others (Pathologic Tumor status grade, tumor grade, gender, ethnicity, alcohol consumption) dropping in importance. This is partially due to variance from non-treatment variables being incorporated into the imputed treatment variables during imputation.

### RNA expression experiments

Table 2 shows that classifier performance was slightly higher (though not significantly so) with the dimensionality-reduced dataset. The best-performing classifier overall appears to be the CIRF, which was middling in runtime. A drastic difference in evaluation runtime is observed (as expected) between the Full RNA feature set (20,541 predictors) and the SPCA feature set (20 predictors). With both feature sets, the non-standard RF variants required more compute time and computational resources than the standard RF classification procedure .

**Fig. 4:**
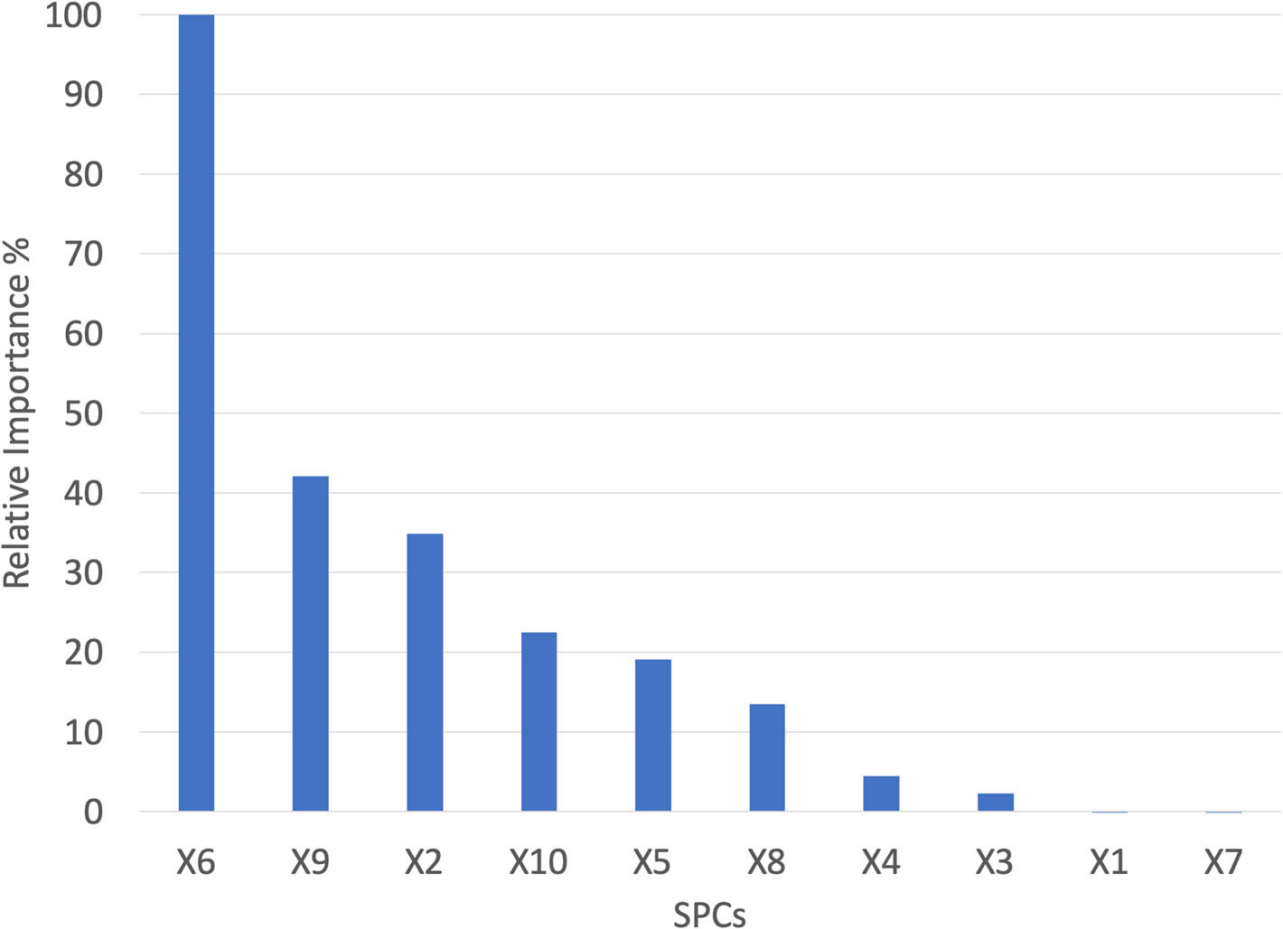
SPC Conditional Importance Values. Relative conditional variable importance values for the 10 SPCs, labeled X1–10. In cases where a very low importance is reported for an SPC, its effect on classifier performance is negligible

Explained variances for all SPCs are reported in Table 3. Considering Fig. 4, SPC X6 is favored most by the conditional inference importance metric. SPCs X9 and X2 are the next-highest ranked. X7, X1, and X3 were the least important variables to the CIRF classifier, indicating they had little-to-no effect on classification performance. The genes composing these six SPC features were selected for further examination via GOEA (see Table 4). It is worth noting that within the gene sets constituting the SPCs, many repeats of genes and gene families are present. This is an artifact of gene family co-expression and the tendency of SPCA to focus on genes with high variance.

**Table 3:** SPC Explained Variances

SPC	Percent Explained Variance
X1^a^	53.84%
X2 ^a^	9.43%
X3 ^a^	9.19%
X4	5.31%
X5	3.14%
X6 ^a^	2.27%
X7 ^a^	2.04%
X8	1.67%
X9 ^a^	1.24%
X10	0.93%

Explained variances for the sparse principal components. The 10 SPCs account for 89.05% of the original data’s variance. ^a^ denotes SPCs chosen for further analysis based on variable importance (see Fig. 4)

**Table 4:** RNA SPCA Enriched GO Terms

SPC	Contributing Genes (Gene Name|GeneID)	Enriched GO Biological Processes
X6	ADAM6|8755, FBP4|2167, FN1|2335, GAPDH|2597, KRT13|3860, KRT16|3868, KRT17|3872, LOC96610|96,610	Cornification
X2	COL1A1|1277, COL1A2|1278, COL3A1|1281, FN1|2335, KRT13|3860, KRT14|3861, KRT16|3868, KRT17|3872, KRT5|3852, KRT6A|3853, SPARC|6678	Cornification^a^, keratinocyte differentiation, wound healing, cell-substrate junction assembly^a^, collagen fibril organization^a^
X9	ACTB|60, ADAM6|8755, COL1A1|1277, COL1A2|1278, FN1|2335, LAMC2|3918, TGFBI|7045	Skin morphogenesis, protein heterotrimerization, platelet activation^a^, cell junction assembly, cell junction organization, extracellular matrix organization, extracellular structure organization, blood vessel development^a^, cell adhesion
X7	ADAM6|8755, FABP4|2167, KRT16|3868, KRT17|3872, KRT5|3852, KRT6B|3854, LOC96610|96,610, PI3|5266	Cornification^a^, programmed cell death, cell death, keratinization, skin development
X1	KRT14|3861, KRT16|3868, KRT17|3872, KRT5|3852, KRT6A|3853, KRT6B|3854, KRT6C|286,887, S100A9|6280	Cornification^a^, intermediate filament cytoskeleton organization^a^, cell death, hair cycle
X3	COL1A1|1277, COL1A2|1278, COL3A1|1281, KRT13|3860, KRT14|3861, KRT16|3868, KRT17|3872, KRT5|3852, KRT6A|3853, KRT6B|3854, KRT6C|286,887, SFN|2810	Cornification^a^, multicellular organism development, intermediate filament cytoskeleton organization^a^, collagen fibril organization

SPC gene sets listing both gene names and Entrez gene IDs; PANTHER annotation terms found to be enriched in each of the SPC gene sets. Annotation terms are reported in increasing order of *p*-value, with all *p* < 0.001. ^a^ indicates some lower level hierarchical terms omitted for brevity

## Discussion

### Imputation evaluation

With imputation, classifier performance is not negatively affected, which is expected based on other studies using the MICE imputation technique [17, 31]. Increases in variable importance after imputation indicate that the treatment variables more effectively predict patient outcomes after application of MICE. It is worth noting that imputation incorporates variance from the features used for imputation into the imputed variables, likely boosting their apparent importance and detracting from the importance of features integral to imputation. Because importance is calculated with a random forest, the importance changes in non-imputed variables might indicate that the bias introduced by MICE imputation to the treatment variables modifies the landscape of variable interactions to a high enough degree that the feature selection within trees is affected.

### RNA expression experiments

For this prediction problem, the dimensionality-reduced features (SPCs) allow comparable classifier performance while drastically reducing runtime and necessary computation. Though not quantified here, memory requirements were also much lower for the dimensionality-reduced data. Additionally, this reduction allowed us to identify gene set candidates for GOEA. In both important and not-important SPCs, the GO term “cornification” is found, indicating that this biological process is related to high-variance genes in this dataset. Terms found only in the high-importance SPC gene sets are related to cell motility (cell adhesion, extracellular interactions), immune response, cell growth, and blood vessel development. Activity of genes involved in these processes could be indicative of a cancer’s ability to survive, grow, and metastasize, suggesting that these SPCA transformed RNA data contain useful information about underlying relationships between solid- tumor expression and two-year recurrence-free survival.

### Conclusions

In modern oncological research, TCGA datasets present significant large data analysis challenges, from clinical parameter sparsity to high dimensionality. Facing these problems requires significant preprocessing and machine learning modeling to uncover new knowledge. A multivariate imputation method (MICE), SPCA dimensionality reduction, and an SPC-focused GOEA are presented in the context of TCGA-HNSC clinical and RNA expression variables to improve usability of data for future HNSCC decision support.

vAs others [17, 31] have found, MICE is an effective practical method for imputing data, though introduction of some bias is very likely. In this case variable importance of imputed features was improved, while the importance measures of other variables were reduced through interaction effects and addition of bias to the imputed variables. Most importantly, the imputation provided a complete set of treatment variables to incorporate into our models, furthering our ability to evaluate the effectiveness of potential biomarkers in later analyses.

Unsupervised transformation of RNA expression data via SPCA was extremely useful in improving interpretability of survival models and biomarker identification, by limiting the number of genes contributing to each principal component and allowing for a more nuanced examination of the underlying biological processes. The biological processes found to be associated with only high-importance SPCs may be useful in future feature vselection for biomarker discovery. Additionally, the SPCA functioned well as a dimensionality reduction technique, as the dimensionality reduced features allowed for significantly lower computation time without significantly affecting classifier performance. From the literature and these analyses, unsupervised transformations of RNA expression data seem a viable option for future integration of molecular data into HNSCC clinical predictive models.

Future work will consider the effect of clinical imputation on models also utilizing molecular data, both with SPCA transformations and other unsupervised feature transformations methods such as denoising autoencoders. Additionally, biomarker evaluation will be expanded to directly consider right-censored survival.

## Data Availability

The TCGA data analyzed in the current study are available in the National Cancer Institute Genomic Data Commons Data Portal, https://portal.gdc.cancer.gov/. The pre- and post- imputation clinical data, the full and transformed RNA data, and our code for running these analyses are available via GitHub at https://github.com/mrendleman/MachineLearningTCGAHNSC-BINF/.

## Abbreviations

AUC: Area under the Receiver Operating Characteristic Curve
CIRF: Conditional inference random forest
CV: Cross-validation
GO: Gene ontology
GOEA: Gene ontology enrichment analysis
HNSCC: Head and neck squamous cell carcinoma
HPV: Human papillomavirus
MICE: Multivariate imputation by chained equations
RF: Random Forest
SPC: Sparse principal component
SPCA: Sparse principal component analysis
TCGA: The Cancer Genome Atlas
TCGA-HNSC: TCGA dataset for HNSCC
WSRF: Weighted subspace random forest
